# Great Northern Festival Dinner

**Published:** 1923-12

**Authors:** 


### ROYAL NORTHERN DINNER.

A FESTIVAL dinner of the Royal Northern Group of Hospitals was held at the Mansion House on November 21, Lord Beatty presiding. Proposing "Success to the Hospital," he said that the Royal Northern Hospital had a remarkable record. In 67 years it had grown from the humble origin of one bed in a single room in a little side street in North London to its present great efficiency and size. The group, explained Lord Beatty, comprised the Royal Northern Hospital, Holloway, the Royal Chest Hospital, City Road, the Hospital of Recovery, Southgate, and the Reckitt Convalescent Home at Clacton. Referring to the last named, Lord Beatty said that he knew from experience that the air of the North Sea had a quality for bracing up in the shortest time a patient even *in extremis*.

The group served a population of 1,000,000 in North London, and of its 375 beds, 70 were closed owing to lack of funds. Money was badly needed for endowment—the hospital obtained only four per cent. of its income from endowment, whereas many ancient institutions obtained as much as sixty-six per cent. from this source. Lord Northampton, chairman of the hospital, had generously defrayed the expenses of the dinner, and on arrival that evening he (Lord Beatty) had been presented with a cheque for 1,000 guineas collected by Mr. Isaacs at the Victoria Club. Lord Northampton said that their expenditure for the year was about £85,000, and the deficit was £15,000. At the close of the evening, the secretary, Mr. Gilbert Panter, announced that subscriptions amounted to £11,643 14s. 7d.

